# Development of a compassion-focused and contextual behavioural environment and validation of the Therapeutic Environment Scales (TESS)

**DOI:** 10.1192/pb.bp.114.048736

**Published:** 2016-02

**Authors:** David Veale, Sarah Miles, Iona Naismith, Maria Pieta, Paul Gilbert

**Affiliations:** 1 Institute of Psychiatry, Psychology & Neuroscience, King's College London; 2 University of Derby, UK

## Abstract

**Aims and method** The aims of the study were to develop a scale sensitive enough to measure the interpersonal processes within a therapeutic environment, and to explore whether the new scale was sensitive enough to detect differences between settings, including a community based on compassionate mind and contextual behaviourism. The Therapeutic Environment Scales (TESS) were validated with 81 participants in three different settings: a specialist service for anxiety disorders, a specialist in-patient ward and a psychodynamic therapeutic community.

**Results** TESS was found to be reliable and valid. Significant differences were seen between the services on the dimensions of compassion, belongingness, feeling safe, positive reinforcement of members' acts of courage, extinction and accommodation of unhelpful behaviours, inconsistency and high expressed emotion. These processes were over time associated with improved outcomes on a specialist service for anxiety disorders.

**Clinical implications** The TESS offers a first step in exploring important interpersonal relationships in therapeutic environments and communities. An environment based on a compassionate mind and contextual behaviourism offers promise for the running of a therapeutic community.

Long-term rehabilitation wards, residential units and therapeutic communities continue to serve a wide range of populations with chronic mental health difficulties. However, the interpersonal processes that facilitate change within a therapeutic environment are under-researched. A central unanswered question is how such services can enhance their outcomes through their environment. An area of concern at our anxiety disorders residential unit was whether we were harnessing the potential of the community to enhance outcomes. Therefore, we focused on the quality and style of relationships within the community to optimise the therapeutic environment. This makes sense, especially given the power of affiliative relationships to change a whole range of processes including physiological processes.^1-3^

We developed a model that builds on attachment theory of group psychodynamic therapeutic communities.^4^ The model consisted of an evolutionary and compassion-focused approach,^5^ which incorporates the principles of learning theory and functional analytical psychotherapy.^6^ In brief, the environment created is one that incorporates compassion (a sensitivity to the suffering of others and a deep commitment to relieve and prevent that suffering), connectedness to other members and regulation of potentially damaging high expressed emotion or punishment by shaming. The term ‘members’ refers to both residents/patients and staff in a community. Four aspects are therefore facilitated:

1. being genuinely and authentically compassionate to one another
2. being open and trusting of compassion from others
3. developing self-compassion rooted in deepening self-awareness and empathic commitment to try to help oneself
4. members (staff and residents) are encouraged to be aware of each other's problems and acts of courage and respond with natural reinforcement to create a safe, collaborative and supportive environment for all.

No relevant scale exists for measuring such an environment. Therefore, the first aim of the current study was to develop a scale that measures the interpersonal processes of such an environment. The second aim was to explore whether the new scale was sensitive enough to detect differences between settings. This is a report on the first attempt at setting up a community with a compassion-focused and contextual behavioural environment. It was hypothesised that such an environment would demonstrate significant differences between a specialist anxiety disorders unit, a group psychodynamic therapeutic community and an in-patient ward, and that the change in the milieu on the anxiety disorders unit would be associated over time with an improved outcome.

## Method

The study had several stages. First, the new self-report scale to measure people's experiences of core interpersonal domains and processes in a therapeutic environment was validated. The use of the scale was then explored in three different settings in a group cohort design. The main outcome measure for the treatment of obsessive–compulsive disorder (OCD) was also compared before and after developing the new environment at a specialist anxiety disorders service.^4^ Ethical approval for the study was gained from Harrow Research Ethics Committee in London (reference: 11/LO/1418).

### Participants

Participants were recruited from three adult mental health settings (Table 1). The first was the anxiety disorders residential unit (ADRU) at Bethlem Royal Hospital. ADRU is a national specialist service providing intensive cognitive–behavioural therapy (CBT) for people with severe treatment-refractory anxiety disorders. The service has 16 beds and is only staffed during the daytime. The average stay is 12 weeks. Forty-two participants, mainly with severe OCD, were recruited.

**Table 1:** Comparison of demographic variables between participants (*n* = 81) by setting

Variable	ADRU (*n* = 42)	Therapeutic community (*n* = 25)	In-patient unit (*n* = 14)	ADRU *v.* therapeutic community ADRU *v.* in-patient unit In-patient unit *v.* therapeutic community
Age, years: mean (s.d.)	37.7 (14.7)	41.1 (8.0)	45.4 (14.2)	H_(2)_ = 4.47, *P* = 0.107

Weeks at the unit, mean (s.d.)	9.8 (2.9)	79.5 (69.4)	18.6 (13.9)	H_(2)_ = 42.57, *P*<0.001
				*U* = 1019.00, *Z* = 6.42, *P*<0.001, d = 2.53
				*U* = 360.50, *Z* = 1.27, *P* = 0.206, d = 0.34
				*U* = 307.00, *Z* = 3.87, *P*<0.001, d = 1.58

Gender, *n* (%)				
Male	20 (47.6)	5 (20.0)	1 (7.1)	Fisher's exact test *P* = 0.006
Female	22 (52.4)	20 (80.0)	13 (92.9)	

Main diagnosis, *n* (%)				
OCD	33 (78.6)			
Body dysmorphic disorder	7 (16.7)			Fisher's exact test *P*<0.001
Social phobia	1 (2.4)	1 (4.0)		
General anxiety disorder	1 (2.4)			
Depression		2 (8.0)	8 (57.1)	
Bipolar disorder		1 (4.0)	1 (7.1)	
Borderline personality disorder		21 (84.0)	5 (35.7)	

Good Milieu Index score, mean (s.d.)	19.6 (4.3)	16.4 (2.7)	14.7 (3.0)	H_(2)_ = 23.34, *P*<0.001
				*U* = 237.00, *Z* = −3.75, *P*<0.001, d = 1.03
				*U* = 87.50, *Z* = −3.93, *P*<0.001, d = 1.23
				*U* = 227.00, *Z* = 1.54, *P* = 0.125, d = 0.51

EssenCES subscales	3.3 (0.7)	2.5 (0.7)	3.1 (0.8)	H_(2)_ = 18.64, *P*<0.001
Patient's Cohesion, mean (s.d.)				*U* = 193.50, *Z* = −4.32, *P*<0.001, d = 1.24
				*U* = 230.50, *Z* = −1.21, *P* = 0.225, d = 0.33
				*U* = 103.50, *Z* = −2.10, *P* = 0.035, d = 0.71
Experienced Safety, mean (s.d.)	3.5 (0.6)	2.4 (0.7)	3.3 (0.4)	H_(2)_ = 33.60, *P*<0.001
				*U* = 104.00, *Z* = −5.49, *P*<0.001, *d* = 1.81
				*U* = 182.00, *Z* = −2.14, *P* = 0.032, d = 0.60
				*U* = 57.00, *Z* = −3.47, *P*<0.001, d = 1.34
Therapeutic Hold, mean (s.d.)	3.4 (0.7)	2.3 (0.7)	2.4 (0.9)	H_(2)_ = 26.92, *P*<0.001
				*U* = 152.00, *Z* = −4.86, *P*<0.001, d = 1.48
				*U* = 121.50, *Z* = −3.28, *P* = 0.001, d = 0.98
				*U* = 175.00, *Z* = 0.00, *P* = 1.00, d = 0.00

Total EssenCES score, mean (s.d.)	3.4(0.6)	2.4(0.6)	2.9 (0.3)	H_(2)_ = 37.05, *P*<0.001
				*U* = 114.00, *Z* = −5.33, *P*<0.001, d = 1.72
				*U* = 89.00, *Z* = −3.89, *P*<0.001, d = 1.22
				*U* = 67.50, *Z* = −3.15, *P* = 0.001, d = 1.17

ADRU, anxiety disorders residential unit; OCD, obsessive–compulsive disorder.

The second setting was a specialist in-patient unit, a national specialist service for affective and personality disorders. It had 18 beds and the average stay was 6 months. Fourteen participants with either recurrent depression or personality disorder were recruited. The unit was unexpectedly closed for financial reasons during the study, which limited the numbers recruited.

The third setting was a traditional psychodynamic therapeutic community. Members attend at least 3 days a week and there is an average of 25 members attending over a 2-year programme. Twenty-five participants, mainly with borderline personality disorder, were recruited.

### Measures

#### Therapeutic Environment Scales (TESS)

The TESS was developed with the aim of measuring the occurrence of various interpersonal processes in the therapeutic environment. It is theoretically driven, with nine subscales of interpersonal behaviour that may influence the environment. These domains were chosen after a review of the literature as having the most evidence for promotion of safeness and courage to change one's behaviour.^4^ The subscales include:

1. positive reinforcement by others at the time of an act of courage (which is defined as difficult or anxiety-provoking and is related to the person's goals)
2. extinction of their own unhelpful behaviours (e.g. self-harming, ritualising) depending on the response of others
3. communication with honesty, openness and genuineness
4. feeling safe with others to express needs or to try out new behaviours
5. belongingness and shared purpose with a responsibility to others
6. compassion (defined as being sensitive to distress in others with a deep commitment to try to relieve it).

There are three negative subscales:

7 inconsistency in responses by others
8 accommodation of unhelpful behaviours and taking over responsibility by others
9 high expressed emotion by others.

The TESS has three main sections: part 1 examines the respondent's experience with staff, part 2 asks about their experience with non-staff members (other residents or patients), and part 3 assesses processes that do not relate to interactions within the community but rather to the individual's own behaviour (goal-setting and tasks, participating in structured activity, democracy and ability to influence the environment, keeping to and questioning boundaries). Responses on all items are given on a 7-point Likert scale (1 ‘strongly disagree’ to 7 ‘strongly agree’). Negative items are reverse-scored. A mean score is then generated for each subscale. All items refer specifically to the past week. Items were generated by consideration of the theoretical model. The questionnaire and scoring details may be downloaded from the online data supplement to this paper.

#### The Essen Climate Evaluation Schema (EssenCES)

The EssenCES^7^ was originally validated for assessing the social and therapeutic atmosphere of a forensic psychiatric unit. It is a self-report scale composed of three five-item subscales: ‘Experienced safety (*v.* threat of violence)’, ‘Patients’ cohesion and mutual support’ (measuring peer support) and ‘Therapeutic hold and support’ (relationship with staff). The range for the total score is 0 to 60.

#### The Good Milieu Index (GMI)

The GMI^8^ is a five-item self-report scale validated for measuring general satisfaction with aspects of the therapeutic milieu: the setting, the staff, the other patients, the programme and their improvement. The items give a total score ranging from 5 to 25. Higher scores reflect higher satisfaction.

#### The Yale–Brown Obsessive–Compulsive Scale (Y-BOCS)

The Y-BOCS^9^ is a ten-item clinician-rated scale that measures severity of obsessive and compulsive symptoms. A total score for the measure ranges from 0 to 40. Higher scores denote greater symptomatology.

### Procedure

#### Creating a compassion-focused and contextual behavioural environment

During the research period, staff at ADRU attended advanced training workshops in compassion-focused therapy and functional analytical psychotherapy on the unit. The emphasis was on helping members to gain a psychological understanding of one another's behaviour within an evolutionary and developmental formulation. Members were encouraged to look out for acts of courage and efforts towards change. Residents shared their goals with others in community meetings (e.g. a behavioural experiment or exposure task that had been agreed) and on a daily message board. The aim was for members to respond naturally with compassion as soon as they noticed a resident's acts of courage and efforts at improvement (‘positive reinforcement’). This approach was coupled with a compassionate mind and tolerance of unhelpful behaviours that affected fellow residents (‘extinction’). Residents were taught self- and other-compassion in weekly groups delivered by staff with relevant imagery exercises and mindfulness. They were taught to communicate with one another in an honest and compassionate manner without being judgemental or critical, and without accommodating each other's problems. All members were encouraged to support each resident to follow their valued directions with structured activity and tasks that supported their goals. As in a traditional therapeutic community, residents were given more responsibility to run it. They had self-allocated roles, including looking after communal areas, dealing with porters, cleaners and caterers directly, and helping recruit new staff.

#### Administering the TESS

Participants within each setting were asked for consent before completing the questionnaires. They were offered the choice of completing the TESS again 3 days after the initial completion. All participants who completed the questionnaires were given a high street shopping voucher to thank them for their time on the project.

#### Statistical analysis

Comparisons of demographic characteristics for all 81 participants were conducted using Chi-square comparisons, Kruskal-Wallis comparisons and *post hoc* Mann–Whitney *U*-tests. Mixed analysis of variance was applied to compare Y-BOCS outcome scores of residents at ADRU before the new environment was introduced with outcomes after the change. TESS subscale scores were compared across the three settings using Kruskal-Wallis tests and *post hoc* Mann–Whitney *U*-tests. All preliminary inferential analyses had a significance value of α = 0.05 and *post hoc* tests used the Bonferroni adjusted significance value of α = 0.017.

Internal consistency of the TESS was examined by calculating Cronbach's alpha (α) for each of the subscales. Subscales whose Cronbach's α value could be rounded up to 0.70 or more are generally considered to have acceptable internal consistency.^8,10^ Items were deleted from subscales where their removal caused an increase in Cronbach's alpha to 0.70 or more. Test-retest reliability of the total scale was analysed on 15 participants who completed the TESS twice. Spearman's rho correlations were conducted to determine the association between scores from the two administrations, occurring 3 days apart. A 3-day hiatus period was chosen as this was short enough to minimise chances of genuine changes in the environment, but long enough to avoid recall effects. Convergent validity of the TESS was measured using Spearman's rho correlations between each subscale with outcome measures of therapeutic environments. Average rankings of TESS scores for relationships with staff and other non-staff members were compared using Wilcoxon signed-rank test comparisons for each setting.

## Results

### Demographic characteristics

Table 1 shows demographic baseline characteristics of participants. At both the in-patient unit and the therapeutic community, significantly higher proportions of participants were females. There were no significant differences between the mean ages of participants. As expected, there were significant differences in the main diagnoses and lengths of residents' stays across the settings.

### Internal consistency of the TESS

The Cronbach's alpha for the TESS subscales ranged from 0.68 to 0.92, indicating that 20 out of 22 of the subscales had acceptable internal reliability (Table DS1 in Online data supplement). One subscale (‘Activity’) had a Cronbach's α of 0.63 and was considered acceptable as there are only four items. One subscale (‘Boundaries’, α = 0.57) was not improved by deleting one item and was therefore removed from further analysis.

### Test re-test reliability

Repeat reliability of the TESS was analysed based on a subgroup of 15 participants completing the scale twice (Table DS2). The TESS scores showed good stability over the 3-day interval. All but 2 of the 22 subscales were correlated to a significant level and Spearman's rho values ranged from r_s_ = 0.54, *P*<0.05 to r_s_ = 0.95, *P*<0.01. The ‘Inconsistency in behaviour’ subscales from part 2 of the TESS and ‘High expressed emotion’ from part 1 were not significantly correlated over time.

### Convergent validity

The positive reinforcement, extinction, communication, safety, belongingness and compassion TESS subscales were all significantly positively correlated with the GMI and EssenCES total, whether it was for staff or non-staff members (Table DS3). Inconsistency, accommodation and emotional expression were significantly negatively correlated with GMI and EssenCES scores for staff. The high expressed emotion was the only subscale that negatively correlated with the GMI and EssenCES total in non-staff members.

### Comparison on Good Milieu Index

ADRU was rated as scoring significantly higher on the Good Milieu Index than either the in-patient unit or the therapeutic community (Table 1). The GMI scores showed that there were no differences between the therapeutic community and the in-patient ward.

### Comparison on EssenCES

ADRU residents rated the total EssenCES scores as higher than both the therapeutic community and in-patient ward (Table 1). They rated the subscales of patient cohesion, experienced safety and therapeutic hold as significantly higher than the therapeutic community did. The in-patients also rated their patient cohesion and safety as significantly higher than the therapeutic community members did.

### Comparison between staff and non-staff members' experiences within a setting

At ADRU staff members were scored significantly higher than non-staff members for positive reinforcement, extinction, communication, honesty, genuineness, safety and compassion (Table DS7, Fig. DS1). Conversely, staff at ADRU were scored significantly lower for inconsistency, accommodation and high expressed emotion than non-staff members.

Staff scored significantly higher than non-staff members only for extinction and safety at the therapeutic community (Table DS8, Fig. DS2), and higher for inconsistency at the in-patient unit (Table DS9, Fig. DS3). Conversely, staff at the therapeutic community and in-patient units were scored significantly lower for high expressed emotion than were non-staff members. All other subscales measured at both the in-patient unit and therapeutic community did not differ significantly between staff and non-staff.

### Comparison of relationships with staff (TESS part 1) across settings

**Fig. 1:**
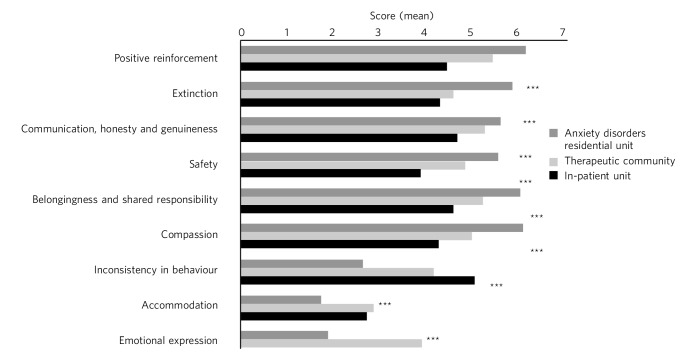
TESS part 1: experience with staff. Comparison between settings. ***P<0.001.

There were significant differences between the settings for all of the relationships with staff scores (Fig. 1, Table DS4). The ADRU scored significantly higher than both the in-patient unit and therapeutic community for positive reinforcement, extinction, safety, belongingness and compassion. ADRU also scored significantly higher than the in-patient unit for communication. ADRU scored significantly lower for measures of accommodation, inconsistency and emotional expression than the therapeutic community, and significantly lower for accommodation and emotional expression than the in-patient unit. There were no significant differences between the in-patient and therapeutic community scores for part 1 of the TESS.

### Comparison of relationships with non-staff members (TESS part 2) across settings

**Fig. 2:**
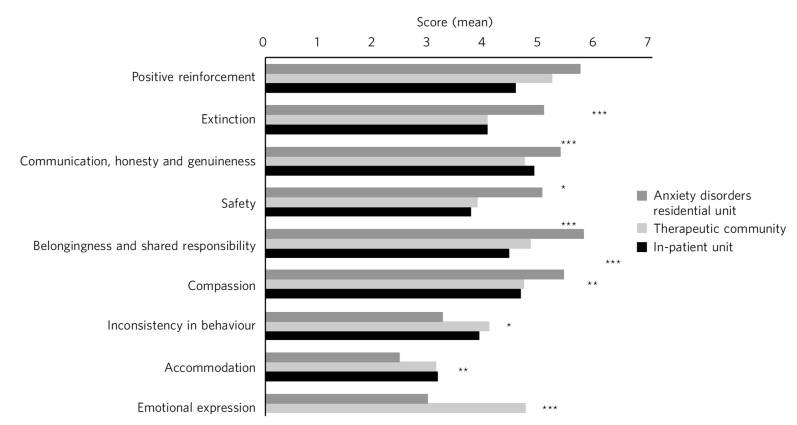
TESS part 2: experience with other members. Comparison between settings. ***P<0.001, **P<0.01, *P<0.05.

All the subscales on the relationship with non-staff members differed significantly across the three settings (Fig. 2, Table DS5). Specifically, ADRU had significantly higher ratings than both the in-patient unit and therapeutic community for positive reinforcement, extinction, safety, belongingness and compassion. ADRU scored significantly higher for communication than the therapeutic community but not the in-patient unit. ADRU had significantly lower ratings for accommodation, inconsistency and emotional expression than both the in-patient unit and therapeutic community. There was no difference between the scores on the in-patient unit and therapeutic community on any of the subscales.

### Comparison of own behaviours scales (TESS part 3) across settings

**Fig. 3:**
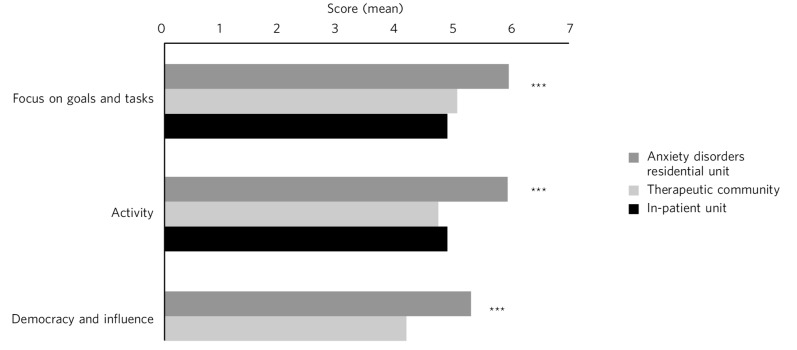
TESS part 3: my own behaviour towards other residents. Comparison between settings. ***P<0.001.

ADRU residents reported significantly higher scores for goals and tasks and democracy than both in-patient and therapeutic community settings. There were no differences between the in-patient unit and therapeutic community (Fig. 3, Table DS6).

### Comparison of ADRU outcomes between 2001–2010 and 2011–2012

**Fig. 4:**
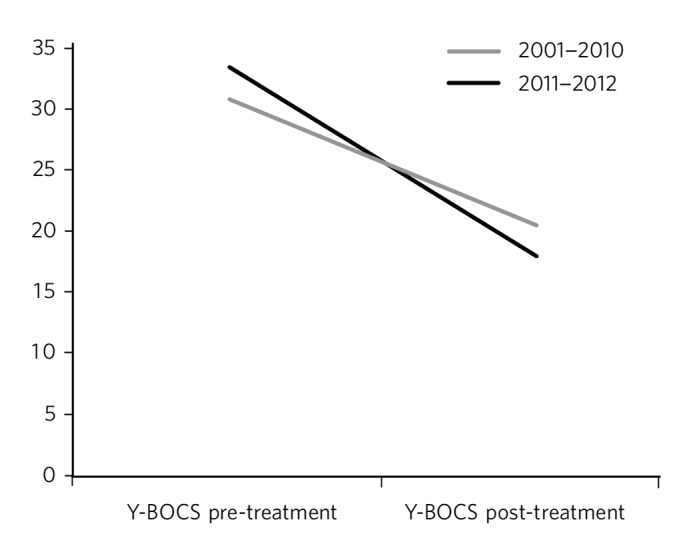
Changes in Yale–Brown Obsessive–Compulsive Scale (Y-BOCS) scores over time for two different time periods at the anxiety disorders residential unit.

After developing a new culture within ADRU, the Y-BOCS outcomes in patients with OCD were significantly improved over time (Fig. 4). In the period 2001–2010, residents' mean Y-BOCS score was 30.4 (s.d.=6.32) at the start of treatment and 20.1 (s.d.=7.52) at the end, whereas during the period of change in 2011–2012, residents' scores were higher at the start of treatment (mean 32.00, s.d.=4.94) and lower by the end (mean 17.06, s.d.=7.60; *F*_(418)_=307.90, *P*<0.001).

## Discussion

The TESS was found to be a valid and reliable scale to ‘take the temperature’ of a therapeutic environment. The subscales were found to have reasonable internal consistency, test-rest reliability and convergent validity. Two subscales of inconsistency and emotional expression subscales had lower test re-test reliability. However, to some extent this is less concerning, as emotional expression and inconsistency are interpersonal processes that one might expect to fluctuate from day to day more than others such as focus on goals. It is recommended that the scales be only administered to all members on the same day. The ‘Boundaries’ subscales were less reliable internally and will require improvement from the current version of the TESS. Of note is that all the subscales were significantly correlated with a good therapeutic milieu except for the subscales of inconsistency, accommodation and high emotional expression which were significantly negatively correlated.

We analysed differences on the TESS within a setting and found that at ADRU, the staff were rated significantly better than non-staff members on all of the subscales. This should be expected as staff receive a higher level of training and experience in the model and are permanent, as opposed to residents who stay for 12 weeks and have both a lower level of training and a primary focus on overcoming their OCD. At the psychodynamic therapeutic community the staff were rated as significantly better than non-staff members for only two subscales (safety and extinction). On the in-patient unit, staff were rated higher on the ‘High expressed emotion’ subscale and lower on the ‘Inconsistency’ subscale (i.e. staff were rated as more inconsistent than non-staff members). This sort of finding would be important for staff in the unit to resolve as it suggests staff conflicts and a lack of feeling safe among members.

We then explored the use of the TESS by comparing different settings. We found that the environment at ADRU scored significantly higher than at the in-patient ward and a group psychodynamic therapeutic community. Members at ADRU agreed more strongly than did the therapeutic community or in-patient unit members that other members were more likely to treat them compassionately, provide them with a sense of belonging and positively reinforce their acts of courage. They felt more supported and safe, which in theory should increase the likelihood that they undertake the necessary behavioural experiments and exposure tasks for improving outcomes. We therefore explored whether outcomes at ADRU improved during the intervention outcomes and found that improved outcomes at ADRU were associated with the period of the intervention.

There are of course limitations to the findings of differences between the settings and improved outcomes. The differences between therapeutic environments are likely to reflect the populations served (e.g. a person with borderline personality disorder may rate their experience of compassion and positive reinforcement by others in a different way to those with an anxiety disorder). Unless one population with the same problem or diagnosis were randomly allocated to different therapeutic environments, we would be unable to conclude that any variations found arose from the different environments. Furthermore, the measure used is based on self-report rather than a behavioural measure by an independent observer. It was also not possible to demonstrate that the changes in ADRU's environment on the TESS led to improved outcomes on the Y-BOCS for OCD. Other factors such as a change in population admitted or other interventions may have contributed to improving outcomes at the unit. However, if the TESS did not find differences between settings or was not associated with better outcomes then one would question the impact of the intervention. The next step would be to determine whether the TESS could moderate an outcome measure after introducing a compassion-focused and contextual behavioural environment in a better designed and controlled study.

### Clinical implications

The implications of our study are that it is possible for a service to measure the interpersonal processes within a therapeutic environment. The scale could act as a measure to ‘take the temperature’ of a therapeutic environment. It may also be used as a potential research tool to determine which interpersonal processes of a community may moderate outcomes.

The TESS can be used freely, and routine monitoring enables staff and non-staff members to identify problems or strengths in a community and guide service changes. Thus, a service might first measure the parameters of its environment at least three times to determine a baseline before introducing changes. Some environments may wish to use part 1 (relationships with staff) only to reduce the length of the scale.

With further development, the scale may be applicable to adolescent and forensic settings or a ‘psychologically informed environment’ (PIE), which is defined as an environment that brings a psychological approach to contexts that may otherwise lack the resources or expertise to run as a formal therapeutic community.^11^

Even if a therapeutic environment in a residential setting is optimised, some residents may then return to an environment of significant criticism, high expressed emotion and accommodation by family members. We are aware of the importance of ensuring that relatives feel involved and valued. In this regard the TESS could be adapted to measuring the family environment. Wherever possible, ADRU staff make home visits and help carers to understand the context of their home environment; if they can, they intervene to develop compassionate responses and a stable and diverse range of natural reinforcers.

Further research is required into the interpersonal processes that promote safeness, connectedness and acts of courage, and whether a service can transform its environment and improve outcomes. For example, a compassion-focused environment recognises that many members are fearful of compassion and react defensively, so working with the fears and avoidance of compassion is a main focus. Second, those with poor empathy or mentalising skills may need to work on them before they can offer compassion to others and feel any genuine interest from others. Third, the environment builds distress tolerance by creating conditions for members to feel the emotion of self-compassion that they try to avoid. Once the model has been refined, we need randomised controlled trials that compare a compassion-focused and contextual behavioural environment with a group psychodynamic therapeutic community for a specific population. In addition, we require research to explore whether training staff in compassion and contextual behavioural models improves not only the environment but also the outcomes. Future research could combine qualitative with quantitative research and offer a before-and-after intervention to transform a service.
